# What Can COVID-19 Teach Us about Using AI in Pandemics?

**DOI:** 10.3390/healthcare8040527

**Published:** 2020-12-01

**Authors:** Krzysztof Laudanski, Gregory Shea, Matthew DiMeglio, Mariana Restrepo, Cassie Solomon

**Affiliations:** 1 Department of Anesthesiology and Critical Care, Hospital of the University of Pennsylvania, Philadelphia, PA 19104, USA; 2 Leonard Davis Institute of Health Economics, University of Pennsylvania, Philadelphia, PA 19104, USA; sheag@wharton.upenn.edu; 3 The Wharton School, University of Pennsylvania, Philadelphia, PA 19104, USA; 4 Department of Internal Medicine, Thomas Jefferson University Hospital, Philadelphia, PA 19107, USA; matthew.dimeglio@jefferson.edu; 5 Department of Biology, University of Pennsylvania, Philadelphia, PA 19104, USA; rmariana@sas.upenn.edu; 6 The New Group Organizational Consulting Inc., Philadelphia, PA 19104, USA; cassie@thenewgroupconsulting.com

**Keywords:** pandemic, COVID-19, artificial intelligence, demand constraints, innovation

## Abstract

The COVID-19 pandemic put significant strain on societies and their resources, with the healthcare system and workers being particularly affected. Artificial Intelligence (AI) offers the unique possibility of improving the response to a pandemic as it emerges and evolves. Here, we utilize the WHO framework of a pandemic evolution to analyze the various AI applications. Specifically, we analyzed AI from the perspective of all five domains of the WHO pandemic response. To effectively review the current scattered literature, we organized a sample of relevant literature from various professional and popular resources. The article concludes with a consideration of AI’s weaknesses as key factors affecting AI in future pandemic preparedness and response.

## 1. Background and Significance

**Figure 1:**
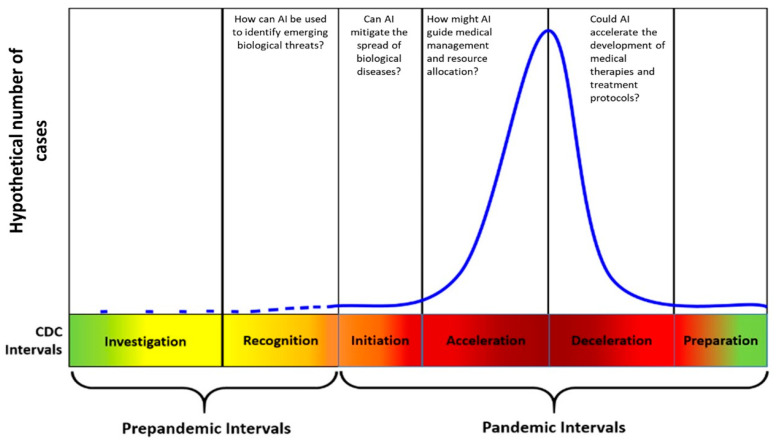
WHO pandemic response phases with distinctive AI applications.

In December 2019, severe acute respiratory syndrome coronavirus 2 (SARS-CoV-2) was isolated from a series of pneumonia cases in Wuhan, China [1]. Extensive international spread has followed since then, causing millions of reported cases and hundreds of thousands of deaths to date [2]. The disease associated with this coronavirus infection (COVID-19) continues to strain healthcare systems and societies at large due to the disease virulence and morbidity [3,4]. History offers examples of pandemics that unfolded similarly to COVID-19, and the World Health Organization (WHO) created a framework describing distinctive intervals of an infectious outbreak (Figure 1) [5,6,7,8]. Initially, the “identification” of transmission of a novel virus, viral strain, or biological organism to humans must be identified. The “recognition” interval follows and features case clusters’ identification globally. “Initiation” comes next and references the development of sustained human transmission. Next, case rates speed up during the “acceleration” interval concomitant with the deployment of several mitigation strategies. These efforts lead to the plateauing and subsequent decrease in case rates, which permits the international community to enter the next pandemic interval, “deceleration.” As case rates finally slow, the international community enters the final interval, “preparation” (for the next pandemic wave) [8].

Artificial intelligence (AI) contains the potential to address all these pandemic challenges, a fact slowly being realized by healthcare stakeholders [9,10,11,12,13,14]. In this manuscript, we will define the AI as algorithms built on mathematical models, themselves continuously and automatically refined through iteration within a “training dataset,” that is, through a set of examples used to increase their prediction capabilities [10]. This article utilizes the WHO pandemic framework to organize consideration of four questions regarding AI’s qualifications in each of the pandemic’s intervals: (1) how can AI be used to identify emerging biological threats? (2) Can AI mitigate the spread of biological diseases? (3) How might AI guide medical management and resource allocation? (4) How might AI accelerate the development of medical therapies and treatment protocols? This article concludes with a consideration of AI’s weaknesses and then three questions about the overall future of AI in healthcare and pandemic response.

## 2. Materials and Methods

The authors employed the WHO framework to inform an Internet search using keywords from each pandemic interval (emergence, recognition, identification, mitigation, deceleration) on the cohort of 866 PubMed manuscripts obtained after combining “artificial intelligence” and “deep learning” with “pandemic” to filter relevant articles published in 2000–2020. Additionally, considerable and appropriate AI writing appears in popular journals due to some of the AI information’s proprietary and policy-related nature. Similar keywords were used in the Google™ search engine. Identified hits were reviewed by one of the authors in terms of relevance to this manuscript. Two other authors verified the information by cross-referencing the findings and searching PubMed with the peer-reviewed manuscripts most relevant to the investigated popular article. The authors believed that combining non-scientific and scientific sources produced a useful breadth of quality references on this topic that is more helpful for this audience than a limited search across peer-reviewed articles. The authors compared non-scientific sources wherever possible with scientific ones as described to achieve satisfactory validation. When independent publications covering similar subjects had no relevant peer-reviewed professional counterpart, the authors looked for the most representative article among the independent publications covering similar topics.

## 3. Results

### 3.1. How Can AI Be Used to Identify Emerging Biological Threats during Investigation and Detection?

Severe acute respiratory syndrome (SARS), influenza virus H1N1, Middle Eastern respiratory virus (MERS), Zika virus, and Ebola have challenged world health in the 21st century to date [1,5,6,7]. The clinical presentations of new illnesses frequently make it difficult to distinguish them from known conditions, even when the potential for transmission is identified [15,16,17,18,19]. Interestingly, the review of the scientific literature revealed little peer-reviewed literature detailing the role of AI in the detection of pandemic emergence [13,17,18,20,21]. However, several technologies emerged, but they were used by private companies [17,18,19,22]. BlueDot, the company inspired by the SARS outbreak, used AI analysis of multiple data points and detected the outbreak in the middle of December 2019 [22]. In this case, analysis of news reports and postings on social media revealed an increase in the use of surgical masks coupled with the emergence of mysterious pneumonia, as did HealthMap [22,23]. Additional confirmation came from Dataminr, another AI company, which combined AI with humans on the ground observation [24]. These examples demonstrate the strength of AI—to sort through an enormous set of data for the clues escaping human analysis [10,25,26,27]. The same examples illustrate the importance of verifying the findings in the process of utilizing the AI [22,24,25]. These particular examples demonstrate that AI and humans augment each other, not being replacements.

The AI relies on a large amount of existing data to train pattern recognition, potentially suggesting that looking for a novel pandemic may not be the best application for AI [10,28]. However, AI can be pre-trained on similar diseases, e.g., by using MERS or SARS data for COVID-19 [9,10,23]. Furthermore, an alternative approach, zero-shot learning, seems to be particularly suitable in pandemic detection training for AI as it allows for learning when almost no clue is possible, but assumptions behind this technique (e.g., variables are independent) may be unrealistic [20,29]. The effectiveness of AI algorithms is challenging to study. The AI algorithms operated by Dataminr or BlueDot are not publicly available and, most likely, not comparable [30]. Other systems are more open in terms of public knowledge of their architecture. The study from 2012 demonstrated no difference in predictive values, yet, the manuscript is relatively old [17]. Lack of insight into how these systems are engineered, or perception of AI being a black box, may be one reason for poor reliance on AI-delivered warnings [9,11]. The industry approach to keep the algorithms hidden under the patent law is not helpful [30,31].

The early detection and forecast of new pandemic emergence provide a time to ramp up supplies, preparedness, and crash research programs. Unfortunately, human decision-makers failed to act according to the AI alert and prior virologists’ warnings [15,16,24]. In other words, AI is but a tool in human hands [10,28,32]. Ignoring the warning from the best tool will render it useless. In stark contrast to ongoing mistrust in AI capabilities, Canada has moved into the “preparation stage” for the next pandemic and signed a contract with Bluedot for an early warning system to run continuously [22,33].

### 3.2. Can AI Forecast and Mitigate the Spread of Biological Diseases during the Initiation and Acceleration of a Pandemic?

Mitigation of a pandemic depends on the ability to identify infected individuals and actual and potential hotspots. BlueDot analyzed the tickets and flights from Wuhan and determined potential hotspots with very high accuracy in December [22]. Soon, other AI engines were forecasting the dynamics of outbreaks in Saudi Arabia, Egypt, Brazil, Canada, India, USA, and African countries [26,33,34,35,36]. The most popular approach in AI design incorporates a long-term short memory-based AI engine utilizing rolling training sets [26,33,37,38,39]. Others used advanced autoregressive integrated moving average [18,35,40]. It remains to be seen which of these AI engines perform with higher sensitivity. The data sources could be numerous and include governmental data, social media, and collateral data from mobile devices or public surveillance systems [17,37,38,41]. The relative scarcity of data for training constrains but does not prohibit AI development and deployment even when resources are limited [42]. Furthermore, the pre-training of AI can help counteract the fact that data are only limited. For example, BlueDot leveraged SARS data [22]. AI can couple existing large databases of living area characteristics (e.g., type of housing, population, and movement of people) with dynamics of the outbreak. Subsequently, AI provides even more accurate prediction of the disease spread, privacy concerns about collecting and sharing this data notwithstanding [22,24,25,42]. AI’s potential to analyze at the level of a city, district, and neighborhood is a powerful feature of this technology [10,25]. The incorporation of contact tracing further augments the power of AI prediction in the spread of a pandemic [23]. The effect of mitigating measures can be studied to identify the most effective ones [43,44,45]. However, the accuracy of AI predictions varies in part with how well its pre-training aligns with actual events [38,46]. Of course, inappropriate predictions may lead to inaccurate estimation of the resources leading to misallocation of resources and inhibit pandemic mitigation [38,46]. Developing AI’s predictive and analytical upside will, in turn, provide a robust base for overcoming arguably the most significant barrier to the use of AI, namely, the trust of humans and their political and social representatives.

### 3.3. How Might AI Guide Medical Management and Resource Allocation during Acceleration and Deceleration of a Pandemic?

Insufficient COVID-19 testing has significantly hampered diagnosing affected individuals. Radiologic alternatives to serology and viral tests were proposed to link emerging clinical symptoms to the COVID-19 infection. Some data suggest that radiological pieces of evidence are more robust [47,48,49]. Consequently, the Italian Society of Medical and Interventional Radiology suggested the use of AI to estimate prognosis for hospitalized patients, albeit stopping short of recommending the use of AI-analyzed CT images as a screening test [50]. Upside notwithstanding, neither the CDC nor the American College of Radiology (ACR) mentioned AI-guided image analysis and its potential to discern image features beyond traditional human-guided analysis [51]. Several other models for AI-augmented analysis of portable X-rays were proposed due to the higher accessibility of X-ray machines vs. CT scanners [52,53,54]. For several years, AI has been applied to image analysis, but even more significant progress was made when large, publicly available data were made available to the scientific community [12]. COVID-Net is an AI-based algorithm trained on the COVIDx database of over 13,000 images [55]. Authors had previously trained AI on pre-existing data sets before acquiring a specific COVID-19 data set, which enabled them to demonstrate AI’s capacity to diagnose COVID-19 by image analysis [50,56,57,58,59]. The sheer size of the data is notable, but researchers’ efforts to provide open, transparent, and validated tools for physicians are trailblazing and remarkable. Often, AI designers do not stress these features, which leads to limited reach and low trust in AI-driven protocols [60,61]. This may be of particular concern if non-radiological data such as chatbots, phone information, and web informatics are used to train AI [62,63].

AI-driven triage is a somewhat controversial application of this technology. However, the mismatch between the ability of a healthcare provider system to deliver health services and demands generated by patient influx is one of the top determinants of mortality in COVID-19. Hospitalized patients, and especially intensive care unit patients, necessitate very resource-intensive treatment for a prolonged period. When the healthcare system reaches saturation, healthcare providers have to make fast decisions regarding which incoming patients will receive care [64,65,66,67]. The COVID-19 pandemic precipitated several approaches to handling the mismatch, one being a framework based on a scoring system with modification for specific consideration, an example being the Italian College of Anesthesia, Resuscitation, and Intensive Care (SIAARTI) guidelines for the criteria that doctors should follow under COVID-19 [67]. This system follows the logic of several similar efforts outside pandemic situations. The United Network for Organ Sharing point systems provides an additional example of using quality-adjusted life-years and disability-adjusted life-years [68,69]. Others have advocated military-type triage based on age [70]. Using an AI-augmented triage protocol could also reduce the enormous stress related to making choices, which are repetitive and deeply challenging healthcare workers during the pandemic with significant ramifications during the pandemic and post-pandemic. Even slightly aiding a provider in deciding about resource allocation might well result in fewer and less intense cases of posttraumatic stress disorder (PTSD) among care providers, especially physicians [71,72]. Less extreme situations in emergency physicians and trauma surgeons result in PTSD [73,74]. Numerous predictive models of COVID-19 prognosis in various individuals based on AI-driven algorithms have been designed and published [75,76,77,78,79,80]. Their ability to distinguish between favorable outcomes and demise is significantly accurate. A few of them were implemented to test their suggestions in real life, a fact that leaves unaddressed concerns about dataset impartiality and concomitant ethical concerns about the implication of AI—driven decisions [68,81,82]. Both concerns can be addressed [61,83]. Firstly, allocating medical care in the absence of the best possible medical information hardly qualifies as the most ethical approach, unless one wishes to argue that willful ignorance qualifies as an ethical (or legal) defense. Second, AI can incorporate ethical standards into its algorithms [31,64,66]. Of course, incorporation does not guarantee that everyone will view the recommended decision as the most ethical possible.

A pandemic such as the COVID-19 one necessitates matching the illness to the level of care in large numbers of patients and the capacity to respond swiftly should their condition deteriorate [3,4]. Current healthcare systems often experience difficulty in detecting rapid changes in health conditions and struggle to adjust care accordingly. In the case of the COVID-19 pandemic, the presenting situation has been growing more complex as the healthcare system has been adding several remote, non-traditional locations, often employing biosensors, apps, or telemedicine [14,82,84]. Supervising care required by such a dynamic disease across heterogeneous care environments placed on care providers additional and complex demands. In other words, a physician need not, for example, rely solely on patient age, but on a rapidly evolving, data-driven prediction of the usefulness of resources in the successful treatment of any given patient [14,65,85]. In telemedicine, AI-driven algorithms have been introduced to score patient severity [86]. These protocols are relatively mature and augment physician decisions’ quality by accounting for multiple factors not readily available to a caregiver. In the specific case of the current pandemic, Stanford University and the University of Colorado adopted systems for COVID-19 patients trying to predict which patients would deteriorate [87]. The operationalization of these AI-driven algorithms in some critical care situations has demonstrated their effectiveness, but regulatory agencies need to discover a fast-track system for their approval [11]. In a protracted pandemic, AI provides the unrealized potential to allow a healthcare system to adapt more quickly to illness severity, patient by patient, while better protecting its workforce’s mental and emotional well-being.

Patient with COVID-19 are very resource-consuming [1,88]. They require sedation, supportive medication, nutrition, care, and ongoing attention while on supportive respiratory care [4]. The associated demand for medications, durable supplies, expendable items, space, and various types of personnel can produce costly and dangerous bottlenecks [89]. This demand pushes the limits of the existing equipment, including PPE. To date, the response has appeared decentralized and uncoordinated, with locales and nations competing for the same limited resources [89]. Govindan et al. suggest using a decision support system with several factors to guide healthcare resource allocation. The next step is the use of AI to analyze the data [14,85]. AI can couple outbreak data with measures of potential demand and direct the supplies more efficiently by directing resources into anticipated hotspots before full-blown local or national crises emerge as well as adjust for individual care [90,91].

Better predictions about a pandemic’s course enable more effective deployment of healthcare and societal resources [64,92]. Assessment of the likelihood of patient’s survival during initial triage and hospitalization may augment the allocation of healthcare assets on the level of a ward, hospital, or larger geographical area. Triage and outcome prediction are very controversial areas of AI application with the medical community and public unprepared for these applications [77,93]. AI can improve resource allocation and help assess the countries’ preparedness by taking into account the dynamic of the spread and available resources, factors frequently minimized in classic epidemiological models [94].

### 3.4. How Might AI Accelerate the Development of Medical Therapies and Treatment Protocols?

The COVID-19 pandemic has resulted in at least three significant types of AI contributions in the development of science-based treatment of COVID-19.

First, machine learning has helped scientists search through the overwhelming amount of research produced about COVID-19 to inform treatment. For example, the Allen Institute for AI, partnering with several research organizations, created the “COVID-19 Open Research Dataset” (CORD-19) which contains over 44,000 scholarly articles about COVID-19 and SARS-CoV-2, updated daily and machine-readable [95]. A similarly curated dataset, LitCOVID, is available via the NIH, but it is manually curated [96].

Secondly, AI played a significant, if largely unrecognized, role in drug development during SARS by suggesting therapeutic compounds even before the emergence of COVID-19 [21,97]. Regarding COVID-19, it can deliver compound selection clues superior to the initial recommendations of Plaquenil or azithromycin to treat COVID-19, i.e., the recommendations based on underpowered, anecdotal, and methodologically troublesome studies [88]. Early on, an AI application identified 78 novel small molecules as candidates to test for rapid testing as potential treatment compounds. A European group screened the existing compounds suggesting possible treatments [98,99,100]. In this manner, AI offers researchers a novel tool to accelerate selection of the existing medications and the development of original molecules. Such identification does not replace the need for clinical trials, but it can focus clinical trials on the most promising therapeutic compounds.

Furthermore, AI may aid in repurposing existing medications for COVID-19 treatment and thus leverage the advantages of the existing approval use: fewer regulatory hurdles and rapid trial cycle [98]. BenevolentAI, one of several groups leveraging AI, engaged in precisely this kind of targeted discovery and trial process, identified baricitinib as a leading candidate for COVID-19 treatment [101]. This compound underwent a promising if limited clinical trial in Italy and has now entered US clinical trials with Eli Lilly and the National Institute of Allergy and Infectious Diseases.

Thirdly, AI can aid rapid acceleration of the development of an effective vaccine. In the US, the Vaccine Research Institute began turning away from traditional vaccine development in 2018, employing instead newer technologies using either DNA or messenger RNA that could potentially work for multiple viruses [102]. Academic institutions followed suit in the wake of COVID-19. Having these platforms available offers the possibility of shortening the development of a vaccine from 20 months to just over 3 months. Moderna had developed eight mRNA vaccines for a variety of viruses and used AI to choose the most promising therapeutic options in the fight against COVID-19 [102,103].

In short, AI can delve deeply and broadly into what humans know in the service of focusing attention on where to delve more deeply and, as importantly, where not to waste valuable time [104,105] on care protocols or potential compounds and vaccines.

### 3.5. Weaknesses of AI

AI carries the possibility of refined impartiality, a key contributor to its potential acceptance. However, “garbage in / garbage out” highlights the real possibility of embedding human errors and biases in AI For example, the Correctional Offender Management Profiling for Alternative Sanctions (COMPAS) system, an algorithm designed to aid the judicial system, replicated common biases and errors [106]. Only the most careful attention will allow us to make those errors and biases visible so that they can be recognized and addressed [107,108].

Healthcare providers are underrepresented in AI development despite considerable expressed interest [10]. Such underrepresentation will likely lead to several weaknesses: more reluctant acceptance thereof by caregivers, particularly by physicians, and less infusion of traditional medical values into its development [109]. Unfortunately, physicians have limited opportunities during their education to familiarize themselves with AI and the related concepts. Healthcare providers need far more education in AI development and use to understand and facilitate its development and application.

## 4. Final Points

The strength of artificial intelligence comes down to its ability to synthesize a vast amount of data quickly and in a way that humans simply cannot. In a pandemic, data emerge rapidly, proving too voluminous, too varied, and too fast-changing for humans to process into information as quickly as AI can (Table 1). It can more quickly determine which variables determine recovery and which appear irrelevant for a specific patient [10]. Additionally, AI can sort through the effects of practice biases such as the amount of ventilation pressure employed, a practice that varies by country, and so decrease the variation in the decisions made by individual healthcare providers [10]. In short, AI offers the potential to support faster development of clinical protocols for a new and evolving disease such as COVID-19.

Not surprisingly, the notion of incorporating AI decision-making in healthcare stirs considerable controversy, including about responsibility and ethics [13,32]. However, the point here is not that AI will make more and better decisions, but rather that it could facilitate more targeted and ethical decision-making. AI will provide a consistency of application and can, under human supervision, serve to broaden and explicate ethical considerations before disasters such as pandemics strike and necessitate all too profound choices. People could take any of a number of approaches to creating standards to govern development and supervision of AI, be it for healthcare or for autonomous vehicles [31,68,69,110].

In closing, the authors highlight three sets of questions worthy of careful exploration. First, who is responsible for AI in healthcare? The hospital system? The creators of any given AI application? If AI achieves the level of self-programming, who carries responsibility for its actions from then onward? Second, who should own AI? Currently, many private entities conduct much of AI development [30]. Does the fact that AI in healthcare will likely include triage and other life and death decisions argue sufficiently for more universal ownership or at least governance? Does the power of AI argue for a definition of “universal” as nothing less than global? Third, if AI can become independent and self-developing, then should it?

## Figures and Tables

**Table 1:** The promise and peril of using AI in pandemics.

Key Questions	The Promise of AI	The Peril of AI	References
How can AI be used to identify emerging biological threats?	○ Early detection of the leading indicators	○ Need for prompt human validation and response	[1,9,10,11,12,15,17,26,63,64,65,66,67]
Can AI mitigate the spread of biological diseases and guide early treatment?	○ Contact tracing and aggregation feeding prediction of contagion spread ○ Rapid evaluation of treatment options based on prior similar events	○ Privacy and appropriateness of predictive modeling	[1,11,12,68]
How might AI guide medical management and resource allocation?	○ Image analysis-driven diagnosis of disease existence, severity, and prognosis ○ Resource allocation informed by ongoing data-based determination of the likely medical outcome ○ Reduce stress on medical personnel ○ Sophisticated and developing analysis of optimum resource allocation across any chosen variable set (e.g., likely outcome, current and likely resource availability, and probable near-term demand)	○ Refined analysis of poorly refined, incomplete, or biased data ○ Abdication of human responsibility for triage decision-making	[13,14,15,16,17,18,20,25,26,37,69,70,71,72]
How might AI accelerate development of medical therapies and treatment protocols?	○ Rapid identification of treatment and vaccine candidates	○ Erroneous delegation of decisions to AI with insufficient human oversight, e.g., of clinical trials or the role of social disparities	[41,42,43,45,46,48,73]
